# Multiple Recombination Events Drive the Current Genetic Structure of *Xanthomonas perforans* in Florida

**DOI:** 10.3389/fmicb.2019.00448

**Published:** 2019-03-13

**Authors:** Sujan Timilsina, Juliana A. Pereira-Martin, Gerald V. Minsavage, Fernanda Iruegas-Bocardo, Peter Abrahamian, Neha Potnis, Bryan Kolaczkowski, Gary E. Vallad, Erica M. Goss, Jeffrey B. Jones

**Affiliations:** ^1^Department of Plant Pathology, University of Florida, Gainesville, FL, United States; ^2^Gulf Coast Research and Education Center, University of Florida, Gainesville, FL, United States; ^3^Department of Entomology and Plant Pathology, Auburn University, Auburn, AL, United States; ^4^Microbiology and Cell Science, University of Florida, Gainesville, FL, United States; ^5^Emerging Pathogens Institute, University of Florida, Gainesville, FL, United States

**Keywords:** core genome multilocus sequence typing, bacterial evolution, recombination, horizontal gene transfer (HGT), *Xanthomonas perforans*, bacterial spot

## Abstract

Prior to the identification of *Xanthomonas perforans* associated with bacterial spot of tomato in 1991, *X. euvesicatoria* was the only known species in Florida. Currently, *X. perforans* is the *Xanthomonas* sp. associated with tomato in Florida. Changes in pathogenic race and sequence alleles over time signify shifts in the dominant *X. perforans* genotype in Florida. We previously reported recombination of *X. perforans* strains with closely related *Xanthomonas* species as a potential driving factor for *X. perforans* evolution. However, the extent of recombination across the *X. perforans* genomes was unknown. We used a core genome multilocus sequence analysis approach to identify conserved genes and evaluated recombination-associated evolution of these genes in *X. perforans*. A total of 1,356 genes were determined to be “core” genes conserved among the 58 *X. perforans* genomes used in the study. Our approach identified three genetic groups of *X. perforans* in Florida based on the principal component analysis (PCA) using core genes. Nucleotide variation in 241 genes defined these groups, that are referred as Phylogenetic-group Defining (PgD) genes. Furthermore, alleles of many of these PgD genes showed 100% sequence identity with *X. euvesicatoria*, suggesting that variation likely has been introduced by recombination at multiple locations throughout the bacterial chromosome. Site-specific recombinase genes along with plasmid mobilization and phage associated genes were observed at different frequencies in the three phylogenetic groups and were associated with clusters of recombinant genes. Our analysis of core genes revealed the extent, source, and mechanisms of recombination events that shaped the current population and genomic structure of *X. perforans* in Florida.

## Introduction

Bacterial pathogens challenge the sustainability and economics of agricultural production. The most damaging bacterial plant pathogens combine rapid evolution with a tendency for emerging strains to spread quickly over long-distances (Carroll et al., 2014). Characterizing bacterial strains associated with disease outbreaks advances our understanding of changes in pathogen populations and geographic distribution of genetic variation as well as the potential to trace the source of outbreaks. Technological advancements in both sequencing and computational tools have facilitated translational research for bacterial disease management via epidemiological and resistance-based approaches in hosts ranging from humans to plants (Köser et al., 2012; Gétaz et al., 2018).

Evolutionary and epidemiological studies of bacterial populations use core genomes, pan-genomes, and intergenic regions to uncover patterns and processes of strain emergence and spread (Biek et al., 2015; McNally et al., 2016; Jibrin et al., 2018). The process of whole genome sequencing followed by gene-by-gene comparisons to identify core genes, which are present in all sampled genomes, expands MLSA (Multilocus Sequence Analysis) from a half-dozen to several hundred or even a thousand genes (Bialek-Davenet et al., 2014). This reproducible approach for phylogenetic comparisons is termed core genome MLSA (cgMLSA) (Maiden and Harrison, 2016; Ghanem et al., 2017; Moura et al., 2017).

The genus *Xanthomonas* is comprised of plant pathogenic bacteria affecting multiple plant hosts. Fresh market tomato production in Florida is severely affected by bacterial spot disease of tomato caused by *Xanthomonas perforans* (Jones et al., 2004; Horvath et al., 2012). Previous studies on *X. perforans* strains isolated from Florida have shown shifts in the bacterial population with regards to species, races, bactericide resistance, bacteriocin production, effector profiles, and phylogenetic groups (Timilsina et al., 2014; Schwartz et al., 2015; Abrahamian et al., 2018). Prior to the initial identification of *X. perforans* in 1991, only tomato race 1 (T1) strains of *Xanthomonas euvesicatoria* were reported on tomato in Florida (Horvath et al., 2012; Timilsina et al., 2016). The first *X. perforans* strains from Florida were identified as tomato race 3 (T3) strains (Jones, 2004; Timilsina et al., 2016). T3 strains carry the functional XopAF (*avrXv3*) and XopJ4 (*avrXv4*) effectors. In 1998, a tomato race 4 (T4) *X. perforans* strain was identified (Minsavage et al., 2003) that lacked a functional XopAF effector. Various surveys and independent isolations over the last two decades determined that T4 *X. perforans* has become the dominant pathogen causing bacterial spot on tomato in Florida (Horvath et al., 2012; Vallad et al., 2013). While selection for widespread copper tolerance in bacteria is expected due to the historical reliance on copper-based bactericides for the management of bacterial spot disease (Vallad et al., 2010), the drivers of tomato race change (in the absence of host resistance), host expansion, and introduction of novel effector genes are less obvious.

We previously identified at least two phylogenetic groups of *X. perforans* in strains isolated from Florida in 2006 and 2012 using MLSA of six housekeeping genes (Timilsina et al., 2014). Among the two groups, group 2 strains appeared recombinant based on the sequences of two housekeeping genes that were identical to *X. euvesicatoria* strain Xe85-10, isolated from pepper (Timilsina et al., 2014). Although *X. perforans* strains are regarded as tomato specific, a group 2 *X. perforans* strain, Xp2010, was isolated from pepper, and other group 2 strains from tomato were shown to cause disease on pepper (Timilsina et al., 2014; Schwartz et al., 2015). The phenotypic and genotypic changes in group 2 strains suggests that the genomic impact of recombination likely extends beyond the few genes we have previously reported (Jibrin et al., 2018).

Phylogenetic methods are commonly applied to the study of bacterial strain ancestry and diversification (Didelot and Falush, 2007). However, most phylogenetic analysis methods assume recombination is absent, and the presence of recombination in the history of a sample can cause incorrect phylogenies. For multilocus sequence analysis of bacterial populations, the tendency has been to remove recombination in order to correctly interpret ancestral relationships for the unrecombined portion of the genome, the “clonal frame” (Wicker et al., 2012; Croucher et al., 2014; Lu et al., 2016). However, considering the ubiquity and impact of recombination on bacterial genetic diversity and evolution, the effect of recombination on phylogenetic relationships should be considered (Didelot and Wilson, 2015; Mostowy et al., 2017). Horizontal gene transfer can expedite evolution and may influence host-specificity in bacteria (Ochman et al., 2000; Yan et al., 2008). Genetic transfer may result in trait convergence due to shared genes acquired by horizontal gene transfer, or lead to the formation of distinct lineages or phylogroups (McNally et al., 2016). Transduction via virus, transformation by donor DNA, and conjugation with the donor are the three mechanisms by which bacteria acquire genetic material (Ochman et al., 2000). The acquisition of genomic DNA can leave specific signals surrounding the introduced genes at the integration sites (Ochman et al., 2000). For example, genomic movement between bacterial species by transduction is limited by phage-host specificity and the events are mediated by mobile DNA vectors observed along with the translocated genomic DNA (Popa et al., 2017).

Our objectives were to determine the extent of recombination in *X. perforans* genomes from Florida strains, identify recombined genes that contribute to the observed population structure in Florida, and evaluate putative mechanisms of genetic transfer of recombined regions. Using a cgMLSA approach, our study provides insights into the extent of recombination and mechanisms of horizontal gene transfer affecting the core genes that constitute the majority of the genomic background of phylogenetically divergent *X. perforans* genomes. The presence of multiple recombination mechanism signals throughout the genome, affecting both core and pathogenicity associated genes, is consistent with high genome plasticity in *X. perforans*. We provide empirical evidence that recombination of core genes has defined the existing phylogenetic groups of *X. perforans* in Florida. The observed genomic patterns appear to be correlated with traits like host-specificity and overall pathogen fitness and indicate that recombination has an extraordinary impact on evolutionary processes in *X. perforans*.

## Materials and Methods

### Bacterial Strains, Genome Assembly, and Genome Similarity

The genomes of 58 *X. perforans* strains isolated from Florida in 1991, 2006, 2012/13, and 2015 were used in this study (Table 1). Draft whole genome sequences of 33 strains, including reference strain Xp91-118, were previously published (Potnis et al., 2011; Schwartz et al., 2015). The remaining 25 *X. perforans* strains collected in 2015 are also publicly available (Supplementary Table 1). The raw Illumina MiSeq 2x250 basepair reads were reassembled using Spades v.3.11 with read error correction and “--careful” switch (Bankevich et al., 2012). The assembled sequences were validated using filter-spades.py^1^ and Bowtie2 was used to align the assembled reads to identify inconsistencies (Langmead and Salzberg, 2012). Pilon (Walker et al., 2014) was used to remove the inconsistencies identified by Bowtie2. The assembled sequences were filtered to remove sections with coverage less than 2 and contig size less than 500 nucleotides. CheckM identified more than 99% genome completeness with less than 0.6% contamination per genome (Parks et al., 2015; Supplementary Table 1). The genomes were annotated using the IMG/JGI platform (Markowitz et al., 2013). Following assembly, pairwise Average Nucleotide Identity (ANI) based on blast was calculated using jSpecies v 1.2.1 (Richter and Rosselló-Móra, 2009).

**Table 1:** List of strains used in this study.

Phylogenetic group	Year	Strains	Source
Group 1	1991	Xp91-118	Potnis et al., 2011
	2006	Xp4B, Xp4-20, Xp5-6, Xp11-2, Xp15-11, Xp18-15	Schwartz et al., 2015
	2012	GEV872, GEV893, GEV904, GEV909, GEV915, GEV917, GEV936, GEV940, GEV968, GEV993, GEV1026	Schwartz et al., 2015
Group 2	2006	Xp3-15, Xp7-12, Xp8-16, Xp9-5, Xp10-13	Schwartz et al., 2015
	2010	Xp2010	
	2012	GEV839, GEV1001, GEV1044, GEV1054, GEV1063	
	2013	TB6, TB9, TB15	
	2015/16	GEV1921, GEV1989, GEV2004, GEV2009, GEV2015, GEV2049, GEV2063, GEV2098, GEV2115, GEV2116, GEV2117, GEV2120, GEV2129, GEV2132, GEV2135	This study
Group 3	2006	Xp17-12	Schwartz et al., 2015
	2015/16	GEV2010, GEV2097, GEV2112, GEV2121, GEV2122, GEV2124, GEV2125, GEV2127, GEV2130, GEV2134	This study


### Pan-Genome Size

For evaluation of the pan-genome of the 58 *X. perforans* strains, all genes were extracted from the 58 *X. perforans* strains using roary (Page et al., 2015) following gene annotation from prokka (Seemann, 2014). The method yielded a total of 7,245 genes. The pan-genome matrix of gene presence/absence in each genome was used as input for a rarefaction analysis to calculate the average number of genes added with each additional genome (Méric et al., 2014). The calculation was randomized by resampling 100 times. The Heaps law function was fitted to the data using the micropan package in R to the rarefaction curve (Tettelin et al., 2008; Snipen and Liliand, 2015). The Heaps law model estimates the parameter alpha. When alpha > 1, this suggests a closed pan genome and saturated sampling of the gene pool, while alpha < 1 suggests an open pan-genome.

### Core Gene Identification and Alignment

The IMG/JGI annotated sequences were used to identify core genes among the 58 genomes. Nucleotide and amino acid sequences of annotated genes were used as input for core gene identification using get_homologues v.2.0.1.9 (Contreras-Moreira and Vinuesa, 2013). Genes present in at least 95% of the genomes and with 75% pairwise alignment coverage were retained. The genes were parsed using python scripts to strictly define core genes as genes present in 100% of the genomes with intact start and stop codons. This approach was taken to limit the core genes to those most likely to be functional, based on genome annotation, in all strains. Genes with multiple copies were also removed. A total of 1,356 genes met the above criteria. Nine genes annotated as functional by the get_homologues built-in annotation algorithm were not annotated by NCBI nor IMG/JGI, but were included in the analysis. The resulting nucleotide sequences of single copy core genes were individually aligned by MAFFT (Katoh and Standley, 2013) using a biopython script (Cock et al., 2009). Individual gene alignments were concatenated using sequence matrix software (Vaidya et al., 2011) to create a circa 1.09 megabases long sequence for each strain.

### Sequence Typing and Gene Mapping

Individual core genes were sequence typed based on nucleotide sequence identity using a python script. Genes with identical sequences were assigned the same number, representing the sequence type. The process was repeated in a loop for all core genes and an output sequence type matrix was generated. This allowed quick comparison of core genes based on allelic variation. Invariable genes were stripped from the matrix to generate a heat map of allelic profiles using the ggplot2 package (Wickham, 2010) in R (R Core Team, 2013). The heat map was color coded to illustrate the allelic patterns for variable core genes, thus providing a genetic fingerprint.

The relative positions of the core genes were mapped based on the complete genome of *X. perforans* Xp91-118 (NCBI accession number: GCA_000192045.3). We used the collated nucleotide sequences of the core genes of Xp91-118 as queries to BLAST (Zhang et al., 2000) against the complete genome. The output was configured to list the start and end positions in the complete genome for all core genes, which were sorted by position using a python script. BRIG (BLAST Ring Image Generator) software v. 0.95 (Alikhan et al., 2011) was used to visualize the positions of individual core genes in Xp91-118.

### Phylogenetic Analysis

Single gene evolution may be different from the evolution of the organism as a whole, particularly when there is horizontal gene transfer (Gogarten and Townsend, 2005). PhyML v.3.1 (Guindon et al., 2010) was used to construct maximum likelihood phylogenetic trees for single gene and concatenated core gene sequences. Nucleotide substitution models were estimated independently for individual genes and selected based on the log likelihood Akaike Information Criterion result calculated using jModelTest2 (Darriba et al., 2012). General time reversible model with gamma distributed rates and invariant sites (GTR+G+I) was identified as the best nucleotide substitution model for the concatenated sequence. Maximum likelihood trees were constructed with 500 bootstrap samples for both concatenated and single genes using the suggested substitution model. ClonalFrameML (Didelot and Wilson, 2015) was used to reconstruct maximum likelihood trees while accounting for recombination. ClonalFrameML calculates R/theta, nu, and 1/delta, which represent the relative rate of recombination to mutation, new polymorphisms introduced from recombination, and the inverse of average tract length of recombination (Didelot and Falush, 2007; Didelot and Wilson, 2015). The three parameters were calculated for all single gene trees and for the concatenated sequence tree (hereafter referred to as core genome tree). Additionally, genomic clustering observed in the phylogenetic trees was confirmed by principal component analysis (PCA). The sequence types of the core genes were used as input to conduct PCA using micropan package in R (Snipen and Liliand, 2015).

### Detecting Genes Driving Phylogenic Relationships

Phylogenetic distances between the unrooted single gene trees and core genome tree, along with the sequence type matrix, were used to determine the genes influencing the core genome tree topology of the 58 strains of *X. perforans*. Congruency of single gene trees to the core genome tree was assessed using Robinson-Foulds (RF) symmetry. This index represents the distance between two phylogenetic trees by evaluating the number of nodes in a tree that are shared with a reference tree (Robinson and Foulds, 1981). We used the core genome tree as the reference tree. The RF symmetry values range between 0 and 1, such that 0 indicates identical tree topology and 1 indicates completely different tree topologies. For example, phylogenetic trees for genes that were identical in nucleotide sequence among all the 58 *X. perforans* strains did not share any nodes with the core genome tree and the RF value was 1. Alternatively, if any nodes in a single gene tree supported a node in the core genome tree, the resulting RF value was less than 1. RF symmetry was calculated using ETE3 Toolkit (Huerta-Cepas et al., 2016) for each single gene tree against the core genome tree to determine the genes that supported some part of the topology of the core genome tree. The sequence types of genes with RF symmetry < 1 were extracted. The variable genes that exhibited RF values < 1 and supported the phylogenetic grouping in the core genome topology are hereafter referred to as Phylogenetic group-Defining (PgD) genes. Maximum likelihood phylogenetic trees were constructed using concatenated sequences of 241 PgD and 1,115 non-PgD genes separately. The total tree length of the two phylogenetic trees were computed using Analysis of Phylogenetics and Evolution (*ape*) package in R (Paradis and Schliep, 2018) to confirm the role of PgD genes in phylogenetic grouping of *X. perforans* in the core genome tree.

### Identifying Recombination Sources and Recombination Mechanisms

We used two methods to determine if PgD genes may have been horizontally transferred. The sequences of the PgD genes were compared to the NCBI sequence database to determine if alleles were shared with other closely related *Xanthomonas* species. We also calculated the relative impact of recombination to mutation on nucleotide substitution using ClonalFrameML. Clusters of genes identified as variable or PgD, particularly those with high recombination values, were identified and their gene neighborhoods and flanking regions were examined. Gene neighborhood regions from representatives of each phylogenetic group were aligned and examined for the presence of genes suggestive of prior transfer events, including features of plasmids, phages, and transposable elements (Chiu and Thomas, 2004). In addition to clusters of core genes, we confirmed the presence of these recombination associated signatures in neighborhood regions of effector genes that were previously suspected to be horizontal transferred (Timilsina et al., 2016).

## Results

### *Xanthomonas perforans* Pan-Genome

A total of 7,245 genes was identified in the pan-genome of 58 *X. perforans* strains and 2,866 genes were considered as core genes by roary (Supplementary Figure 1A). The Heaps law estimate, on the rarefaction curve of the number of new genes identified after randomly adding a genome, suggested an open pan-genome (Supplementary Figure 1B). The estimate for alpha was 0.813, suggesting that additional genes will be found upon sampling more *X. perforans* genomes.

### Core Genome Phylogeny

The get_homologues pipeline identified 2,031 genes as core genes present in at least 95% of the sampled genomes. The variation in the core genes identified from roary and get_homologues is likely due to the two different annotation pipelines used to generate inputs for these programs. Roary used the annotation output from prokka whereas annotation based on Clusters of Orthologous Groups of proteins (COG) downloaded from IMG/JGI were used for the get_homologues platform for core gene extraction. We manually curated these to 1,356 core genes that were present and intact in all 58 *X. perforans* genomes (Supplementary Table 2). Nucleotide sequence comparison revealed that 783 genes were identical among all 58 genomes. At least two allele types were found in the remaining 573 genes (Supplementary Table 3).

**FIGURE 1:**
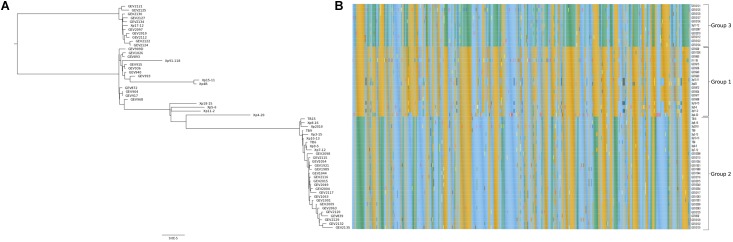
The core genome of Florida *X.* *perforans* strains shows three groups of strains. **(A)** Maximum likelihood phylogeny based on concatenated sequences of 1,356 core genes (∼1.3 Mb) and the branch topology were maintained regardless of the bootstrap support. **(B)** Heat map based on the allelic profile (sequence type) of variable genes (573) found within the core genes.

**FIGURE 2:**
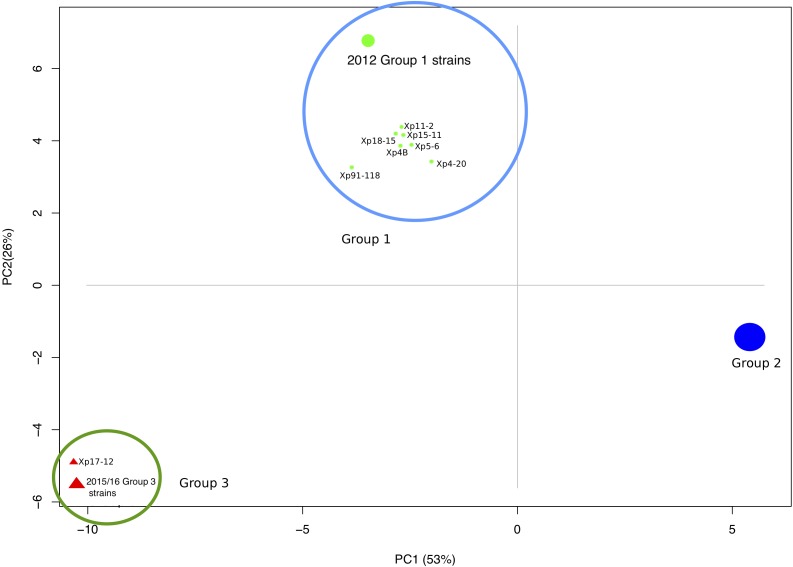
Principal component analysis showing three distinct clusters of *X. perforans* strains from Florida based on the sequence types of 1,356 conserved genes.

The core genome phylogenetic analysis identified a third phylogenetic group in addition to the two previously described groups of *X. perforans* in Florida (Figure 1A). PCA of sequence types of the 1,356 core genes confirmed the three phylogenetic groups (Figure 2). Strain Xp17-12, which was previously considered to be within group 1 (Schwartz et al., 2015), clustered with 10 strains isolated in 2015/16 to form a separate phylogenetic clade that we refer as group 3. Among the 58 strains, 18 strains were designated group 1, 29 strains were designated group 2, and 11 strains were designated group 3. Group 1 is a heterogeneous group that includes 6 strains from 2006 and 11 from 2012 along with the reference tomato race 3 (T3) strain Xp91-118 from 1991. Strains from the 2015/16 season were in group 2 (15 strains) or group 3 (10 strains).

Within groups, the majority of strains shared more than 99.8% pairwise nucleotide sequence identity in the core genome (Supplementary Table 4). Between the groups, identity was reduced to 99.5%. The group 1 strains had relatively lower sequence identities of ∼99.5% in pairwise comparisons, and some strains had group 2 sequence types for several genes as observed in the heatmap and sequence type table (Figure 1B and Supplementary Table 3). Group 2 strains formed a monophyletic group with sequence identity above 99.7% among core genomes except for comparisons with Xp8-16 and Xp2010 (Figure 1A and Supplementary Table 4). Group 3 showed relatively low polymorphism with the majority of strains sharing core genome sequence identity above 99.9%. Average nucleotide identity based on BLAST using the whole genomes of these strains showed similar pairwise sequence identities to core genome comparisons (Supplementary Table 5).

### Variable Core Genes by Phylogenetic Group

**FIGURE 3:**
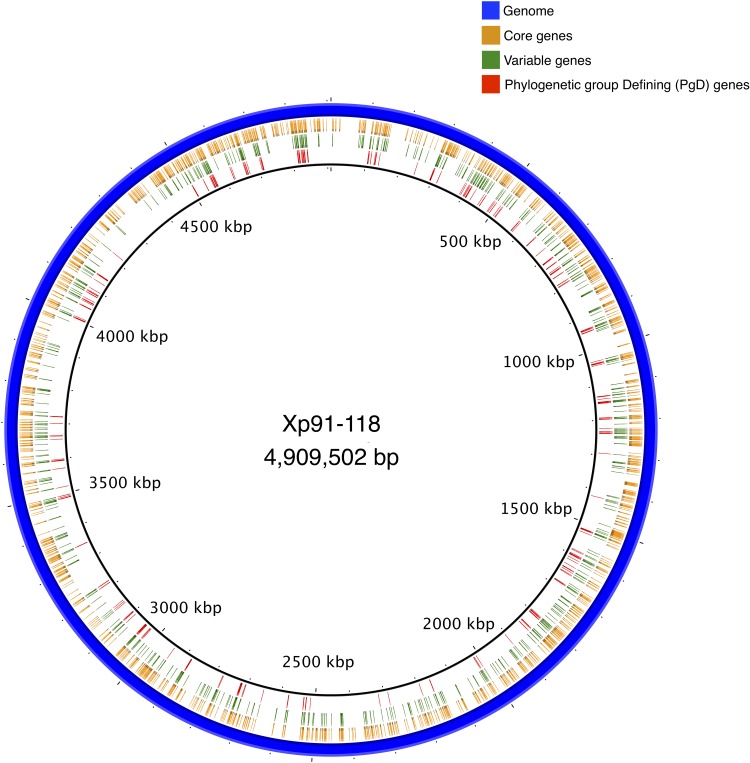
A circular representation of complete genome of Xp91-118 indicating relative positions of core, variable, and phylogenetic group defining (PgD) genes. Clusters of PgD suggest potential recombination hotspots.

Core genes were distributed throughout the Xp91-118 genome (Figure 3). Among the 573 genes that had at least two allele types, referred to as variable genes, allelic variation was often between phylogenetic groups (Figure 1B). Sequences were generally monomorphic or had a single SNP at low frequency within phylogenetic groups. While only 783 genes were monomorphic across the 58 genomes, the number of genes with identical nucleotide sequences within groups were 1124, 1195, and 1239 for groups 1, 2, and 3, respectively.

### Phylogenetic Group Defining Genes

We identified 241 core genes that supported one or more branches of the core genome tree topology that defined the phylogenetic groups (RF < 1). These genes are collectively distinguished as the phylogenetic group defining (PgD) genes as they drive the observed phylogenetic grouping of the 58 strains (Figure 1A and Supplementary Figure 2A). In particular, we observed that these PgD genes carried allele types that were often specific to phylogenetic groups (Figure 1B, Supplementary Table 3, and Supplementary Figure 2A). The annotations of genes identified as PgD are listed in Supplementary Table 2, among which 59 were annotated as hypothetical proteins and 2 as genes with domains of unknown functions. A total of 1,115 single gene trees did not support the core genome tree topology (RF = 1). These included 783 genes that were identical among all the *X. perforans* strains, plus 332 genes that had a variant allele type for at least one strain. The total length of tree based on 241 PgD genes was six times the length of the tree based on the remaining 1,115 core genes, signifying the larger contribution of PgD genes to strain variation (Supplementary Figure 2B). The non-PgD genes contribute to variation shared among a small numbers of strains.

Allele types distinguishing different groups were found in the PgD genes. Among the 241 PgD genes, 96 genes carried an allele specific to group 2 strains (different from the allele in group 1 and 3 strains), and 78 (81%) of those group 2 alleles were identical to *X. euvesicatoria* reference strain Xe85-10 (NCBI Accession no GCA_000009165.1). We found 142 genes with group 3-specific alleles, out of which 64 genes (45%) had alleles identical to *X. euvesicatoria* Xe85-10. An additional five PgD genes with group 3-specific alleles were identical to those of *X. axonopodis* pv. *citrumelo* strain F1 (NCBI Accession no. GCA_000225915.1), which included two hypothetical proteins, a protease modulator HflC, an anti-anti-sigma factor, and a type VI secretion system associated gene. Finally, group 3 alleles of two PgD genes were identical to those of *X. perforans* strain LH3 (NCBI Accession no. GCA_001908855.1), which was isolated from Mauritius in 2010 (Richard et al., 2017). These genes were N-acetyl-gamma-glutamyl-phosphate reductase (AQS75037.1) and aminoglycoside phosphotransferase (AQS78190.1). Therefore, LH3 is the only group 1 strain to contain these group 3 allele types. BLAST searches did not produce exact sequence matches to group 3 alleles for 71 genes. Unique allele types of PgD genes were distributed among group 1 strains. Group 1 strains isolated in 2006 carried specific allele types for 13 PgD genes that were identical to Xe85-10. Xp4-20 and Xp5-6 carried an additional 51and 19 unique allele types, respectively. The remaining four group 1 strains isolated in 2006 (Xp4B, Xp15-11, Xp11-2, and Xp18-15) had specific allele types for 15 additional genes. Group 1 strains isolated in 2012 were homogenous with 15 genes among the PgD genes identical to Xe85-10. Some of the allele types carried by group 1 strains collected in 2006 were identical to group 2 but different from the reference strain Xp91-118. Among all 241 PgD genes, we found three genes that each had three alleles that were specific to group: endopeptidase (AQS77891.1), TonB-dependent siderophore receptor (AQS78913.1), and septum formation protein Maf (AQS76051.1).

Mapping PgD genes to the complete genome of Xp91-118 identified the positions and proximity of these genes (Figure 3). For example, a ∼22 kb region between tryptophan-tRNA ligase (AQS77329.1) and catalase (AQS77307.1), encompassing 16 core genes (14 designated as PgD genes), exhibited diverged haplotypes specific to group 2 strains compared to group 1 and 3 strains. Similarly, an ∼8 kb region, between co-chaperone YbbN (AQS78328.1) and peptidyl-prolyl *cis-trans* isomerase (AQS78967.1) genes, exhibited a distinct haplotype in group 3 strains compared to the other two groups. The overall ratio of changes introduced by recombination relative to mutation in the concatenated core genome tree was estimated to be 16.75 by ClonalFrameML. These values ranged between 0.063 (AQS77927.1) and 184.159 (AQS77019.1) among the individual PgD gene trees (Supplementary Table 2).

### Recombining Genes and Mechanism of Horizontal Gene Transfer

**FIGURE 4:**
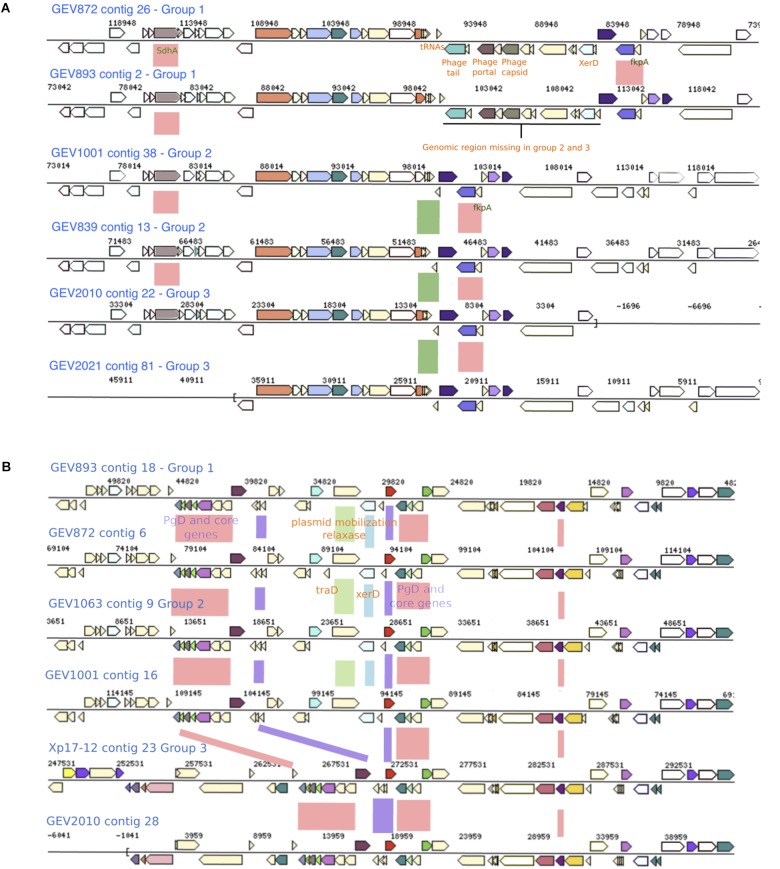
Genomic regions with genes exhibiting group specific alleles and recombination signals. **(A)** A genomic region present in group 1 strains, which contains phage associated genes and is flanked with tRNAs and XerD, is absent in Group 2 and 3 strains. Phylogenetic group defining (PgD) genes are indicated by pink boxes between the alignments with recombinant allele types in group 2 and group 1 2006 strains. The coordinates of this region in Xp91-118 are between 323182 and 325825 **(B)** Group 1 and 2 strains carry XerD, the site specific recombination gene, along with plasmid mobilization and Tra genes (green and blue boxes). This region is missing in Group 3 strains. PgD genes are indicated by pink and purple boxes with recombinant alleles in group 3. The coordinates of this region in Xp91-118 are 1604360-1620009.

Genomic regions acquired via horizontal gene transfer may have signatures of integration associated with different modes of horizontal gene transfer (Ochman et al., 2000). We examined genomes representing each phylogenetic group for signals of recombination flanking clusters of PgD genes. We used two criteria to select genomic regions. First, we were interested in alleles of closely clustered PgD genes that were identical to Xe85-10 indicating gene transfer from *X. euvesicatoria*. Second, we focused on gene trees that exhibited higher ratios of recombination to mutation than the concatenated gene tree. Gene neighborhood comparisons around PgD genes showed the presence of multiple tRNAs, phage-associated and plasmid mobilization genes, along with site specific two-component system *XerC* and *XerD*, which were previously described to be associated with horizontal gene transfer (Aussel et al., 2002). For instance, group 1 strains isolated in 2012 have ∼9.6 kb of phage-associated genes between the tRNA-Leucine and tRNA-Glycine adjacent to the PgD genes AQS75151.1–AQS78555.1 (distinct alleles in group 2 and group 1 strains isolated in 2006). This unique ∼9.6 kb region found only in group 1 strains isolated in 2012 include AAA-domain containing protein, phage major capsid protein, phage portal protein, phage terminase-like protein with HTH domain, and hypothetical proteins. Nucleotide BLAST search in NCBI revealed 98% sequence similarity with only LH3 strain. The site-specific recombinase (*XerD*) gene was observed in all the group 1 strains between the tRNA-Leucine and phage associated genes, suggesting the integration of the unique genomic region was facilitated by bacteriophages (Figure 4A). In general, multiple copies of *XerD*, ranging from four to eight, were observed in all *X. perforans* genomes except for the 1991 reference strain, Xp91-118, which carries only one copy.

Group 3 strains carried 71 PgD genes with unique alleles specific to the group. For example, group 3 alleles of six PgD genes between locus tags AQS76085.1–AQS76101.1 were distinct from the other two groups and were unique among sequences available in NCBI. In group 1 and 2, this genomic region is adjacent to *XerD*, mobile element protein (*MobA*), conjugal transfer protein (*traD*) and phage integrase family protein, suggesting integration via plasmid mobilization and site-specific recombination mechanisms (Figure 4B). The genomic region between the multiple tRNA sites and *XerD* present in group 1 and 2 included several unique hypothetical genes and DNA methyltransferase gene. Furthermore, the genetic variation in group 3 strains indicate the acquisition of novel genomic traits via recombination from multiple donors in addition to *X. euvesicatoria*.

### Recombination Affecting Type III Effectors

**FIGURE 5:**
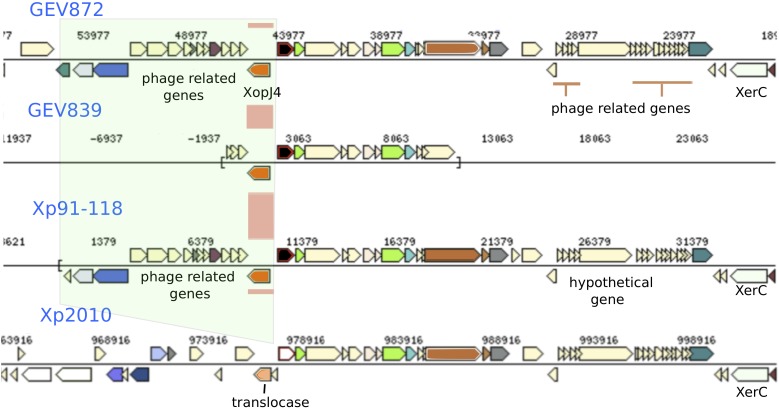
Recombination signals observed surrounding the gene for XopJ4 effector. The XopJ4 gene is flanked by phage associated genes in most strains, including T3 strains Xp91-118. In strain Xp2010, isolated from pepper, the effector gene and phage associated genes were replaced by a putative translocase.

Following the observation of at least two different mechanisms of genetic exchange that were associated with phage transfer and plasmid mobilization in core genes, we examined these signals throughout representative genomes. Interestingly, signatures of horizontal gene transfer were found surrounding effectors that show presence/absence polymorphism among strains and previously predicted to be acquired via recombination. Among the effectors found in bacterial spot causing *X. perforans*, *avrXv4* (*xopJ4*) is found in all tomato pathogenic strains but not found in the strain, Xp2010, isolated from pepper. We found phage associated genes flanking XopJ4 (Figure 5). In Xp2010, both phage associated genes and the *xopJ4* effector are absent. Similarly, the gene coding another XopJ family effector, *avrBsT* (XopJ2), found in the majority of tomato race 4 *X. perforans*, is flanked by genes for type IV secretion system proteins, conjugative transfer, chromosome partitioning protein, and hypothetical proteins (Supplementary Figure 3).

## Discussion

Using a cgMLSA approach, we determined that the *X. perforans* strains isolated in Florida at different times is defined by three groups of strains that are differentiated by hundreds of variable genes, the majority of which appear to be recombinant. All of the strains we analyzed were tomato race 4 *X. perforans* that were collected in the past two decades, except for the 1991 T3 reference strain, Xp91-118, and a pepper strain, Xp2010 (Schwartz et al., 2015). A high-resolution phylogeny of *X. perforans* strains was constructed as well as a core genome fingerprint, which shows allelic variation affecting genes throughout the chromosome. Core genome multilocus sequence analysis allows a holistic comparison of phylogenetic groups and their evolution while minimizing the individual differences between the strains (Maiden et al., 2013; Maiden and Harrison, 2016). Recombination was inferred to be widespread in core genes showing allelic variation with *X. euvesicatoria* strains as a major donor. Furthermore, we observed plasmid and phage associated site-specific recombination mechanisms surrounding clusters of putatively recombinant genes as well as genes that influence host-specificity and pathogen fitness. The open pan-genome further suggests high genomic plasticity in *X. perforans*. We hypothesize that these recombinant strains are epidemic lineages that have emerged in Florida tomato production from a highly recombinogenic source population.

We previously showed two major phylogenetic groups of *X. perforans* in Florida (Timilsina et al., 2014, 2016; Schwartz et al., 2015), but strains collected in the 2015/16 growing season revealed a third monophyletic group of 11 strains. Consistent with recent emergence, it was the most homogenous of the three groups as shown by pairwise nucleotide identity and PCA of sequence types. One strain from this group, Xp17-12, was isolated in 2006 and was previously described as an outlier within group 1 (Schwartz et al., 2015). The relative homogeneity among group 3 strains over time suggests that the 2015/16 group 3 strains were from the same source population. Group 1, which includes strains isolated in 1991, 2006 and 2012, is more heterogeneous than other groups (Figure 1, 2). Group 2 strains have been dominant in Florida since at least 2006 (Timilsina et al., 2016) but appear to be largely clonal (Figure 2). Three genes with alleles specific to each phylogenetic group were observed that could be used for assigning strains to groups to monitor their prevalence in Florida populations going forward. In general, we identified only a quarter of the total genes being shared among all *X. perforans* genomes, which suggests high genome plasticity in the species and is consistent with our finding of an open genome and is in agreement with another study of *X. perforans* (Jibrin et al., 2018).

We inferred recombination to have been the major source of genetic variation in the core genome. Horizontal inheritance of genes from multiple transfer events can obscure ancestral relationships among bacterial strains (Gogarten and Townsend, 2005). Consequently, recombinant loci are often removed from alignments prior to phylogenetic analysis. However, these loci also define the evolution of the recombinant strains (Didelot and Wilson, 2015). Phylogenetic reconstruction without the presence of these potentially recombined PgD genes significantly altered the observed phylogenetic relationships among the *X. perforans* groups (Supplementary Figure 2A). This variation, likely due to recombination from multiple donors, reinforces the necessity to include recombination in bacterial population studies. For group 2, most of the recombinant sequences appeared to have been acquired from *X. euvesicatoria*, which was displaced in Florida by *X. perforans* producing antagonistic bacteriocins against *X. euvesicatoria* (Hert et al., 2005; Timilsina et al., 2016). For group 3, *X. euvesicatoria* was one of multiple donors. The earliest strains of *X. perforans* isolated in Florida belonged to group 1, which had the least recombination signatures relative to the other two groups, as reflected by the copy numbers of site-specific recombinase XerCD genes. Correspondingly, the frequency of recombination observed with *X. euvesicatoria* and other closely related *Xanthomonas* was relatively low in these strains. Although, horizontal gene transfer is largely associated with acquisition of new traits, the tracts of homologous genes potentially acquired from closely related species shows the impact of recombination on the tempo and direction of *X. perforans* genome evolution. Recombination affecting the genetic background of a pathogen can affect niche adaptation, fitness, and microbial competition, and thus the establishment of recombinant strains (Alizon et al., 2009).

Our observations suggest horizontal gene transfer of long fragments of shared recombinant alleles through different genomic vectors. The horizontally introduced genomic fragments can be regulated by the carrying capacity of plasmid or phage vectors (Ochman et al., 2000; Bobay and Ochman, 2017). These two mechanisms of vector associated horizontal gene transfer were clearly visible in our *X. perforans* genomes. Phage and plasmid associated genes were present in the flanking regions of clusters of core genes showing evidence of recombination. Among the three modes of horizontal gene transfer, we were not able to directly identify genomic fragment acquisition via transformation. Several variable genes had variation attributed to recombination, but without any evidence of phage or plasmid associated genes in the flanking regions. These genes may have been transferred via transformation.

Phage mediated gene transfer appears to play a major role in influencing genomic diversity and evolution of *X. perforans*. We found phage genes throughout the genome in regions with potentially recombined core and effector genes. One such example is the XopJ family effector, *avrXv4* (*xopJ4*). XopJ4 is a member of the XopJ effector family, which is similar to the YopJ, serine/threonine acetyltransferase superfamily (Szczesny et al., 2010). A previous study reported that *xopJ4* was conserved in all *X. perforans* strains except for the Xp2010 strain that was isolated from pepper (Schwartz et al., 2015). The *xopJ4* gene was located between phage associated genes. The whole genomic region including phage associated and *XopJ4* genes is missing in the Xp2010 pepper strain. A similar XopJ family effector (98% amino acid identity) in *X. citri* pv. *vignicola* strain CFBP 7112 (Ruh et al., 2017) is also located between phage associated genes. Effector *avrBsT* is another XopJ family effector found in *X. perforans* that is generally associated with plasmids (Ciesiolka et al., 1999; Kay and Bonas, 2009; White et al., 2009). The *avrBsT* effector was not found in *X. perforans* until 1998 (Timilsina et al., 2016). The nucleotide sequence of *avrBsT* in *X. perforans* is identical to that in the more distantly related bacterial spot species, *X. vesicatoria* (Timilsina et al., 2016). An identical allele type of the plasmid-borne *avrBsT* gene in different *Xanthomonas* species, including *X. perforans*, suggests the gene is horizontally transferred across the genus (Timilsina et al., 2014, 2016; Jibrin et al., 2018). The majority of T4 *X. perforans* strains carry *avrBsT* and the gene has been found to provide a competitive advantage to bacterial strains in field conditions (Abrahamian et al., 2018). The variation created by mobile genetic elements in the core and accessory genes could influence host preference and pathogenicity of *X. perforans* strains.

Several PgD genes also showed evidence of plasmid associated horizontal gene transfer. An intriguing pattern that was evident in the genomes was the density of tRNAs and site-specific tyrosine recombinase genes flanking these recombined regions. The two-component site-specific tyrosine recombinase, XerC and XerD, catalyzes crossover and recombination at specific sites (Midonet and Barre, 2015). Site-specific recombination is characterized by cleavage of both DNA strands at two recombination sites that are later joined to new DNA partners without DNA degradation and phosphodiesters hydrolysis (Subramanya et al., 1997). The XerD recombinase works together with XerC, both of which belong to the λ-integrase family. XerD is reported to initiate recombination by strain exchange to form the Holliday junctions and that is reconstructed by XerC (Aussel et al., 2002; Crozat et al., 2015). In *X. perforans* genomes, the site-specific XerD gene was co-located with a plasmid mobilization and transfer (*tra*) gene where a ∼9.6-kb genomic region was present in group 1 genomes but absent in groups 2 and 3. Sequence comparison showed this region is specific to group 1 *X. perforans* strains. Along with XerCD genes, this genomic island was flanked by tRNAs (Figure 4A). The tRNAs serve as a gateway for integration of foreign DNA (Ochman et al., 2000; Williams, 2002). Boyd et al. (2009) reported that tRNA-Arg, -Leu, -Thr, and -Ser were commonly observed insertion sites. The genomic islands introduced by phage or plasmid in *X. perforans* seem to have specific attachment sites, facilitated by tRNAs and site-specific recombinase genes, that altered the core genomes and ultimately shaped the population of *X. perforans* in Florida. These specific sites serve as recombination hotspots in the bacterial genome.

Bacterial spot disease of tomato has posed a series of management challenges in Florida, including the introduction of *X. perforans*. In this study, we have begun to tease apart the genetic mechanisms driving population changes in *X. perforans* since its emergence in 1991, which appear primarily due to phage and plasmid-mediated horizontal gene transfer followed by integration into the chromosome. Our findings indicate rapid genomic evolution in the *X. perforans* population in Florida, which together with our previous findings of extensive recombination in strains from Nigeria and Italy (Jibrin et al., 2018), suggest a pathogen with a high probability of overcoming management practices, i.e., *X. perforans* poses a high “evolutionary risk” (McDonald and Linde, 2002). However, the clonal structure of the Florida population also indicates that a limited number of recombinant genotypes have been introduced to or have successfully established in Florida tomato production. If we could determine the population or populations that are highly recombinogenic, these populations could be specifically managed or movement out of these populations curtailed. For example, recombination events could be occurring in seed sources or other production regions that are not closely connected to Florida, thus exchanging few migrants. Efforts are also needed to understand why *X. perforans* readily recombines when other bacterial phytopathogens, including the bacterial spot pathogen *X. gardneri*, appear highly clonal (Schwartz et al., 2015; Timilsina et al., 2014).

## Data Availability

The datasets generated for this study can be found in NCBI, PRJNA436012.

## Author Contributions

ST, NP, GV, JJ, and EG conceived the project. PA collected additional bacterial strains and provided their genomes. JP-M and FI-B oversaw the genome assembly. GM and ST conducted the sequencing experiments. ST conducted all computational analyses and interpreted with the help of BK, EG, GV, and JJ. ST, EG, JJ, and GV wrote the final manuscript. All authors approved the final manuscript.

## Conflict of Interest Statement

The authors declare that the research was conducted in the absence of any commercial or financial relationships that could be construed as a potential conflict of interest.

## Footnotes

1 https://github.com/drpowell/utils/blob/master/filter-spades.py
