# Supplementary figures and images for: Multiple Recombination Events Drive the Current Genetic Structure of Xanthomonas perforans in Florida
Source: Front Microbiol. 2019 Mar 13;10:448. doi: 10.3389/fmicb.2019.00448 (PMC6425879; doi:10.3389/fmicb.2019.00448)

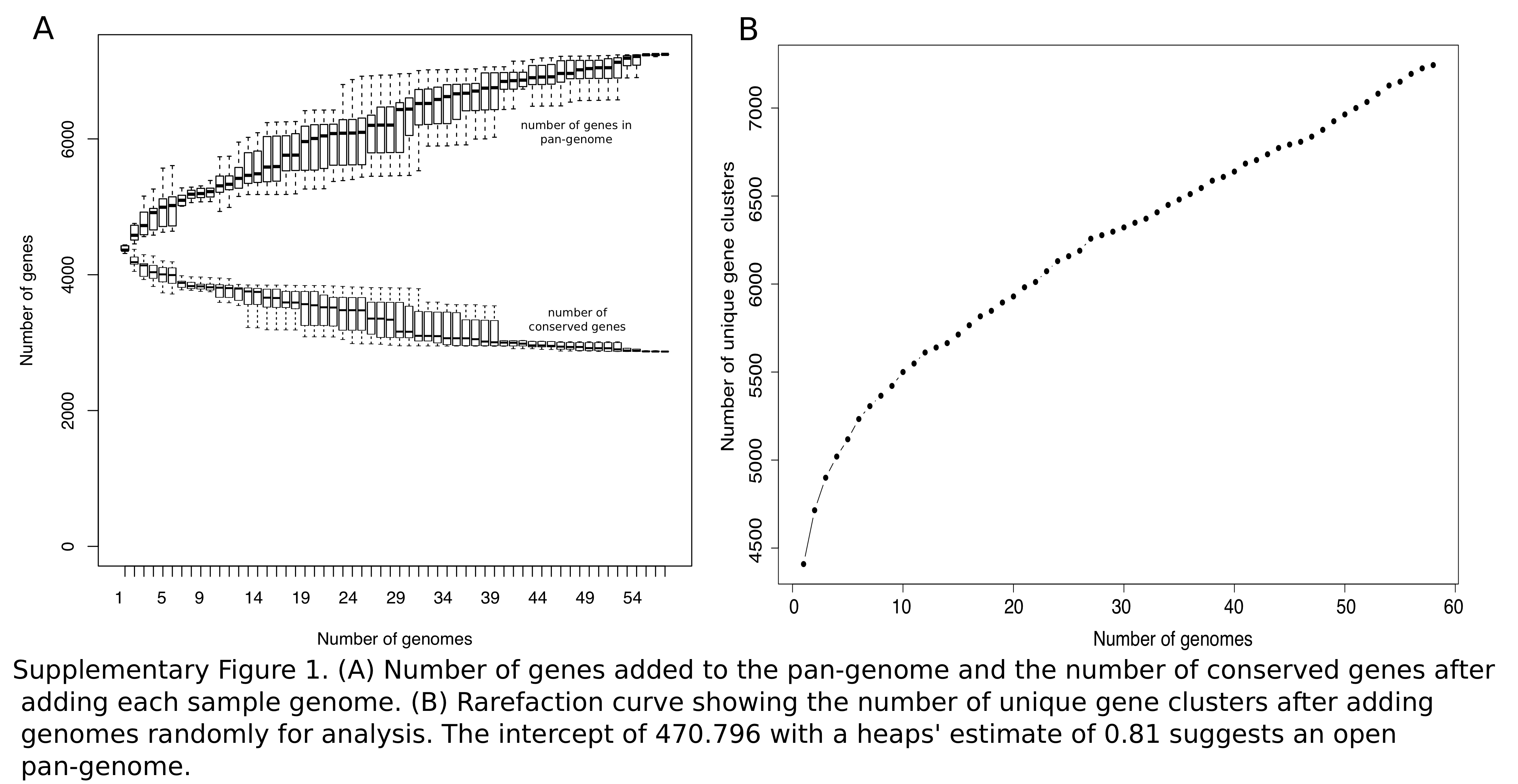

Supplement: Supplementary file 1 [file Image_1.JPEG]

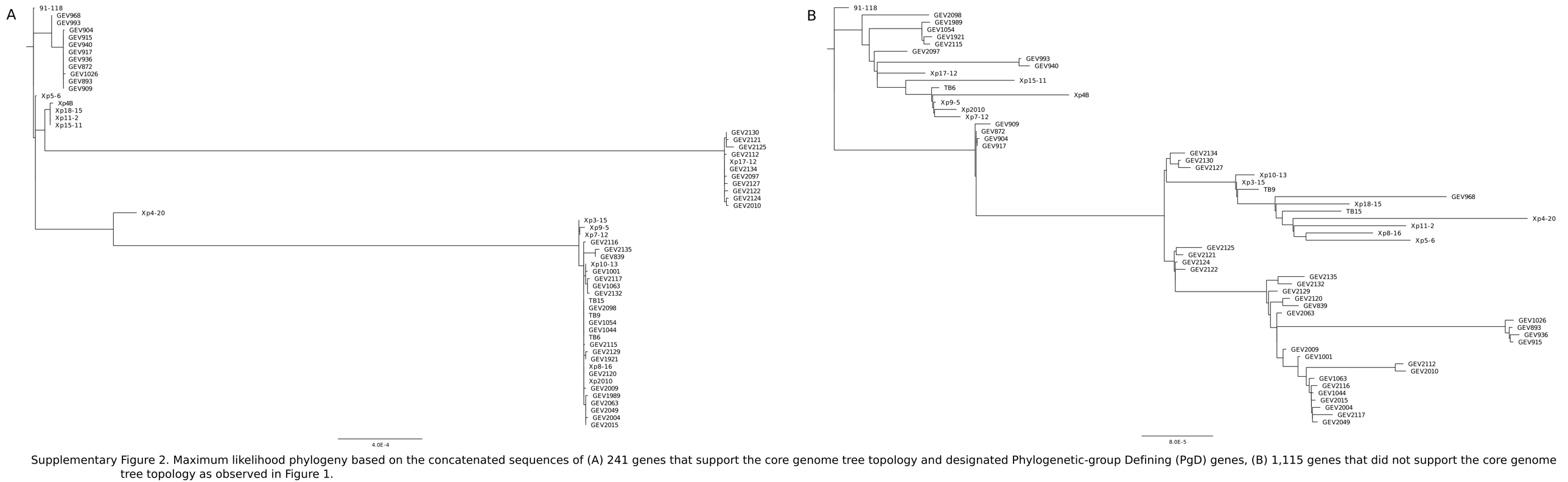

Supplement: Supplementary file 2 [file Image_2.JPEG]

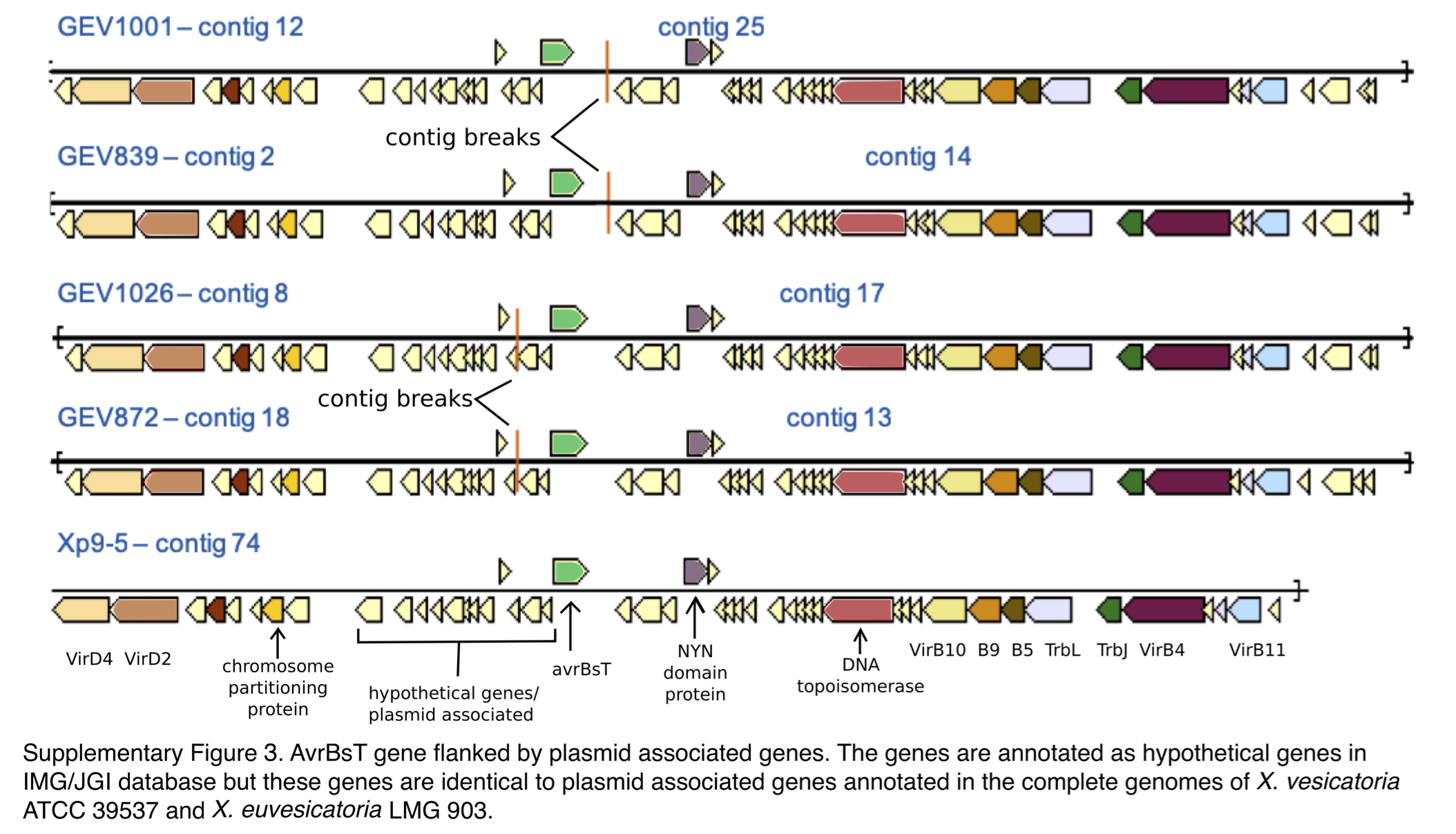

Supplement: Supplementary file 3 [file Image_3.JPEG]
